# Relationship between Gingival Crevicular Fluid Microbiota and Cytokine Profile in Periodontal Host Homeostasis

**DOI:** 10.3389/fmicb.2017.02144

**Published:** 2017-11-01

**Authors:** Jianye Zhou, Yiqing Yao, Kangli Jiao, Jumei Zhang, Xin Zheng, Fang Wu, Xiaopan Hu, Junping Li, Zhanhai Yu, Gaosen Zhang, Nan Jiang, Zhiqiang Li

**Affiliations:** ^1^Department of Prosthodontics, Key Laboratory of Oral Diseases of Gansu Province, Northwest University for Nationalities, Lanzhou, China; ^2^Department of Biological Systems Engineering, Washington State University, Pullman, WA, United States; ^3^Yinchuan Stomatology Hospital, Yinchuan, China; ^4^Xi'an Eighth Hospital, Xi'an, China; ^5^Department of Prosthodontics, School of Stomatology, Lanzhou University, Lanzhou, China; ^6^Department of Soil Research, Cold and Arid Regions Environmental and Engineering Research Institute, Chinese Academy of Sciences, Lanzhou, China; ^7^Department of Applied Soil Biochemistry, Institute of Applied Ecology, Chinese Academy of Sciences, Shenyang, China

**Keywords:** periodontitis, inflammation, cytokines, microbiota, genomics

## Abstract

As potential biomarkers in periodontitis, microbiome, and cytokines have recently been extensively investigated, but combined analyses of the variations between the microbial structure and cytokine composition are rare. The present study aimed to investigate whether there are differences in the combined profile of microbiome and cytokines in individuals with or without periodontitis. The microbiome and cytokine composition in gingival crevicular fluid (GCF) from 16 patients and 15 controls from Jishi Shan (Gansu, China) were analyzed using 454 pyrosequencing and RayBio Quantibody Arrays. The results showed that a higher co-occurrence of genera in periodontitis group compared with the healthy group, as evaluated by Schoener's abundance-based co-occurrence index. C-reactive protein (CRP) was significantly (*P* < 0.05) higher in the GCF of the periodontitis group while interleukin (IL)-8 was significantly (*P* < 0.01) higher in the GCF of the healthy group. The Mantel test revealed a significant concordance between cytokines and microbiota, in the healthy group (Mantel statistic *r* = 0.36, *P* ≤ 0.05) but not in the periodontitis group (Mantel statistic *r* = 0.013, *P* = 0.434). The results were further confirmed by the Procrustes test. Matrix metalloproteinase (MMP)-9, osteoactivin, IL-8, and macrophage inflammatory protein (MIP)-1a were significantly associated with bacterial composition at the phylum, class, order, family, and genus levels. CRP was also associated with bacterial composition at the species level. In conclusion, alterations in the polymicrobial community structure leads to disruption in the healthy correlation between cytokines and microbiomes. This dysbiosis between the microbiota and the immune response could be one of the major etiological mechanisms underlying periodontitis.

## Introduction

Periodontitis is a bacteria-induced inflammatory disease of the oral cavity and has a high worldwide prevalence (Adler et al., 2013), affecting ~50% of the adults and more than 60% of the people over 65 years of age (Chapple et al., 2013). Periodontitis is not exclusively injurious to oral health; indeed, many potential associations between systemic diseases and periodontal disease have been highlighted (Loos, 2006).

The subgingival polymicrobial communities of individuals with and without periodontitis are now well-defined using 16S RNA (Griffen et al., 2012; Abusleme et al., 2013; Kirst et al., 2015) and it has been shown that microbial communities are more closely associated with periodontal disease than any individual bacterial species (Abusleme et al., 2013). Several studies have investigated the association between host immune responses and pathogens such as *Porphyromonas gingivalis* (Jiao et al., 2013; Hajishengallis and Lamont, 2014; Maekawa et al., 2014), *Streptococcus gordonii*, and *T. forsythia* (Kesavalu et al., 2007; Polak et al., 2009; Daep et al., 2011; Kinney et al., 2011; Orth et al., 2011). However, the oral microbiome is not limited to only three species and the complex impact of the various bacteria on periodontal disease is more complicated than the study of single bacterium (He et al., 2015).

It was initially thought that pathogenic bacteria were responsible for periodontitis, but it is now known that the sole presence of the pathogens is not sufficient for tissue destruction and that human microbiome dysbiosis and host inflammatory dysregulation play important roles in periodontal disease (Pihlstrom et al., 2005; Darveau, 2010). Indeed, the host response to bacteria triggers the immune cells and alters the balance between anti-inflammatory and pro-inflammatory cytokines, thereby contributing to disease progression (Darveau, 2010). Nevertheless, the exact mechanisms and contribution of the various factors are still unclear (Human Microbiome Project Consortium, 2012). Several cytokines playing major roles in periodontal disease have been identified (Sexton et al., 2011; Korte and Kinney, 2016) and used as potential biomarkers for periodontitis and periodontal disease progression (Kinney et al., 2014). Interleukin (IL)-1β (Gursoy et al., 2011), IL-6 (Nibali et al., 2012), IL-8 (Lagdive et al., 2013), and C-reactive protein (CRP; Bansal et al., 2014) have been shown to play important roles in periodontal disease. Nevertheless, a comprehensive understanding of the roles of various cytokines and microbial communities in periodontitis is lacking (Casanova and Abel, 2004; Lee and Mazmanian, 2010). Therefore, whole microbial and cytokine profiles should be used for screening and would be more appropriate for predicting periodontitis development, diagnosis, and progression than using single cytokines or bacteria (Gursoy et al., 2011; Kinney et al., 2011; Belstrom et al., 2014).

Therefore, the present study was undertaken to examine the combined profile of microbiome and cytokines among individuals with or without periodontitis. Aboriginal volunteers with similar habits were selected from Meipo Village in Jishi Shan (Gansu, China) in order to minimize the habitat bias on oral microbial communities (Seow, 2012; Wang et al., 2012). To do so, 454 pyrosequencing and RayBio Quantibody Arrays were used (1) to detect the microbiomes and inflammatory cytokines of gingival crevicular fluid (GCF) in volunteers with periodontitis and healthy volunteers, and (2) to analyze the correlations between microbiome composition and inflammatory cytokines. This study extends our understanding of the associated polymicrobial community and cytokines composition in periodontitis.

## Materials and methods

### Subjects and specimen collection

Volunteers were selected from Meipo Village in Jishi Shan (Gansu, China) in September 2013. Subjects who had systemic diseases or immune suppression, had taken antibiotics within the previous 2 months, were pregnant, or were smokers were excluded from this study. Then, 31 subjects, including 16 with and 15 without periodontitis, were recruited (Supplementary Table 1). A complete clinical examination, including medical and dental histories and an intra-oral examination, was performed by a trained examiner. Pocket probing depths (PD) and clinical attachment loss (Shaddox et al., 2011) were measured at six sites (mesiobuccal, buccal, distobuccal, distolingual, lingual, and mesiolingual) according to the Community Periodontal Index (Kinney et al., 2011). Periodontitis patients had PD ≥ 5 mm, CAL ≥ 2 mm, and gingival bleeding on probing (BOP) ≥ 30%; whereas healthy controls had no CAL and probing depths ≤3 mm (information is presented in Supplementary Table 1). The volunteers were fully informed of the research objectives and provided written informed consent after the research was approved by the Ethics Committee of the Northwest University for Nationalities. Studies involving humans conform to the guiding principles of the Declaration of Helsinki.

### GCF extracts

After the supragingival plaque was removed and the target sites dried, four sterile Whatman 3 MM papers were gently inserted for 10 s into the mesio-(one paper), mid-(two papers), and distobuccal orifices (one paper) of the gingival crevice of two molars (first molar or second molar) with the deepest attachment loss for the individuals with periodontitis and of the left upper first molar for the healthy individuals. The four papers were then cut equally from the center line that was vertical to the insertion edge using sterile scissors, transferred to two sterile 1.5-mL tubes separately, and stored at −80°C until use (Sakiyama et al., 2010). No blood was wicked by the sterile paper strips, and microbial genomic DNA and cytokines were both detected in the two tubes, respectively.

### Cytokine detection

GCF samples were eluted from Whatman filter paper in 80 μL of PBS (pH 7.4) containing 0.1 mg/mL phenylmethanesulfonyl fluoride to inhibit the protease activity. Twenty proteins were selected, including CRP, interferon-γ (IFN-γ), IL-1, IL-2, IL-4, IL-6, IL-8, IL-10, IL-12, IL-17, macrophage inflammatory protein-1a (MIP-1a), matrix metalloproteinase (MMP)-9, MMP-13, osteoprotegerin (OPG), osteopontin (OPN), osteoactivin, receptor activator of NF-κB (RANK), transforming growth factor-β1 (TGF-β1), and tumor necrosis factor-α (TNF-α), all of which have been associated with periodontitis (Garlet, 2010; Shaddox et al., 2011). Cytokines were detected using a Human Cytokine Antibody Array Kit (QAH-PDD-1, RayBiotech, Norcross, GA, USA), according to the manufacturer's instructions. Briefly, after blocking, the chips were incubated at room temperature (RT) for 2 h with 50 μL of fresh GCF samples. The chips were then washed and incubated for 2 h with biotin-conjugated antibodies at RT. The chips were washed again and incubated for 1 h with Cy3 equivalent dye-conjugated streptavidin in the dark at room temperature. After additional washes, the signals were visualized using a laser scanner, and the data were extracted using Axon GenePix software.

### DNA extraction, library construction, and sequencing

DNA was extracted from the GCF samples using the MicroElute Genomic DNA Kit (OMEGA, Guangzhou, China), according to the manufacturer's instructions. Firstly, 200 μL of buffer (100 mmoL Tris-HCl (pH 8.0); 25 mmoL EDTA (pH 8.0); 500 mmoL NaCl; 1% SDS; 20 mg/mL lysozyme; 10 mg/mL RNase A) were added to the tubes, and then incubated at 37°C for 30 min. Secondly, 20 μL of protease K (10 mg/mL) were add to the tubes, mixed fully, and incubated at 55°C for 50 min. The DNA was extracted by elution of the columns. To determine the bacterial communities in each GCF sample, the V1-V3 region of the bacterial 16S rRNA gene was amplified by PCR using the primers 8F and 533R (5′-AGA GTT TGA TCC TGG CTC AG-3′ and 5′-TTA CCG CGG CTG CTG GCA C-3′, respectively). Each sample was tagged by adding an index sequence extension at the 5′ end of the forward primers for multiplexing to allow simultaneous analyses of several samples in a single sequencing run. PCR amplification was performed in triplicate using an Eppendorf MasterCycler Ep Gradient Thermal Cycler (Eppendorf, Hauppauge, NY, USA) in a total volume of 25 μL, containing 9 μL of sterilized water, 5 μL of 5 × PCR buffer, 5 μL of 5 × PCR GC high enhancer, 2 μL of dNTPs (2.5 mM), 2 μL of template DNA (200 ng/μL), 0.25 μL of TaKaRa polymerase (5 U/μL), and 1 μL of each primer (10 μM). The thermal cycling conditions were as follows: an initial denaturation at 98°C for 5 min, followed by 27 cycles at 98°C for 30 s, 55°C for 30 s, and 72°C for 60 s, with a final extension at 72°C for 5 min. Following the amplification, 3 μL of PCR product was used for agarose gel (2.0%) detection. The triplicate PCR reactions for each sample preparation were combined and quantified by PicoGreen using a FLUOstar Optima (BMG Labtech, Jena, Germany). The PCR product from each sample was pooled with those of the other samples for one sequencing run. After quality control using Agilent 2100, the pooled mixture was re-quantified with PicoGreen. Pyrosequencing was performed using a Roche GS FLX+ system (Roche, Switzerland).

All sequencing data have been submitted to the Sequence Read Archive (SRA) at NCBI under Project ID SRP118852.

### Sequencing data analysis

All raw sequences were trimmed using the Quantitative Insights Into Microbial Ecology (QIIME) toolkit v.1.5.0 (Caporaso et al., 2010). Next, the sequences were further trimmed by QIIME to exclude the reads that were shorter than 150 bp, harbored an average quality lower than Q25, exhibited a homopolymer longer than 8 bp or more than one mismatch at the 5′-end, or contained any ambiguous bases. Chimeric sequences were identified and removed using UCHIME (Edgar et al., 2011). The operational taxonomic units (OTUs) were clustered with a 97% similarity cut-off using QIIME. Singletons of representative OTUs were removed to reach taxon counts more than 0.001% of the total sequences, and then assigned taxonomically using the Ribosomal Database Project (RDP) classifier (Cole et al., 2005) and the BLAST tool of the Human Oral Microbiome Database (HOMD; http://www.homd.org/; Dewhirst et al., 2010).

Sampling efficiency was evaluated by rarefaction curves using QIIME. The Shannon–Wiener, Simpson (Chao et al., 1992), Chao, and ACE (Chao and Shen, 2003) indices were calculated to measure α-diversity using the Mothur software (http://www.mothur.org/). To estimate β-diversity, the Bray–Curtis (abundance-based) and weighted UniFrac (phylogenetic-based) distance matrices were calculated through QIIME. Comparison of β-diversity between H and P was performed by Constrained Principal Coordinates Analysis (CAP) using the vegan package in R (version 3.1.1). Schoener's index (abundance-based; measuring the co-occurrence probability of two taxa; Schoener, 1992) was computed for each pair of genera across the samples using the R spa package. Heatmaps for evaluating the distribution pattern at the genus level based on Schoener's index in the two groups were drawn using the heatmaps package in R. Principal Component Analysis (PCA) and Redundancy Analysis (RDA) were performed using the vegan package in R. The significance of constraints for PCA and RDA, as well as the significant variables in RDA, were also detected using the vegan package in R. The matrix of correlations and *P*-values for cytokines were calculated using the Hmisc package in R. The correlation matrices between microbes and cytokines were computed by the Mantel test, Procrustes (PROTEST) test, and Correlation matrix using the vegan and CCA package in R, respectively. The correlation coefficients were calculated from the relative abundance of genera, and the significance of correlations was estimated. Linear discriminant analysis Effect Size (LEfSe) was used to determine the taxa that characterized the differences in bacterial composition between the H and P groups. Specifically, the non-parametric factorial Kruskal–Wallis sum-rank test was used to detect taxa whose relative abundance was significantly different, and Linear Discriminant Analysis (LDA) was used to estimate the effect size of each differentially abundant taxon (Giardine et al., 2005; Blankenberg et al., 2010; Goecks et al., 2010).

## Results

### Bacterial communities in GCF of individuals with and without periodontitis

**Figure 1:**
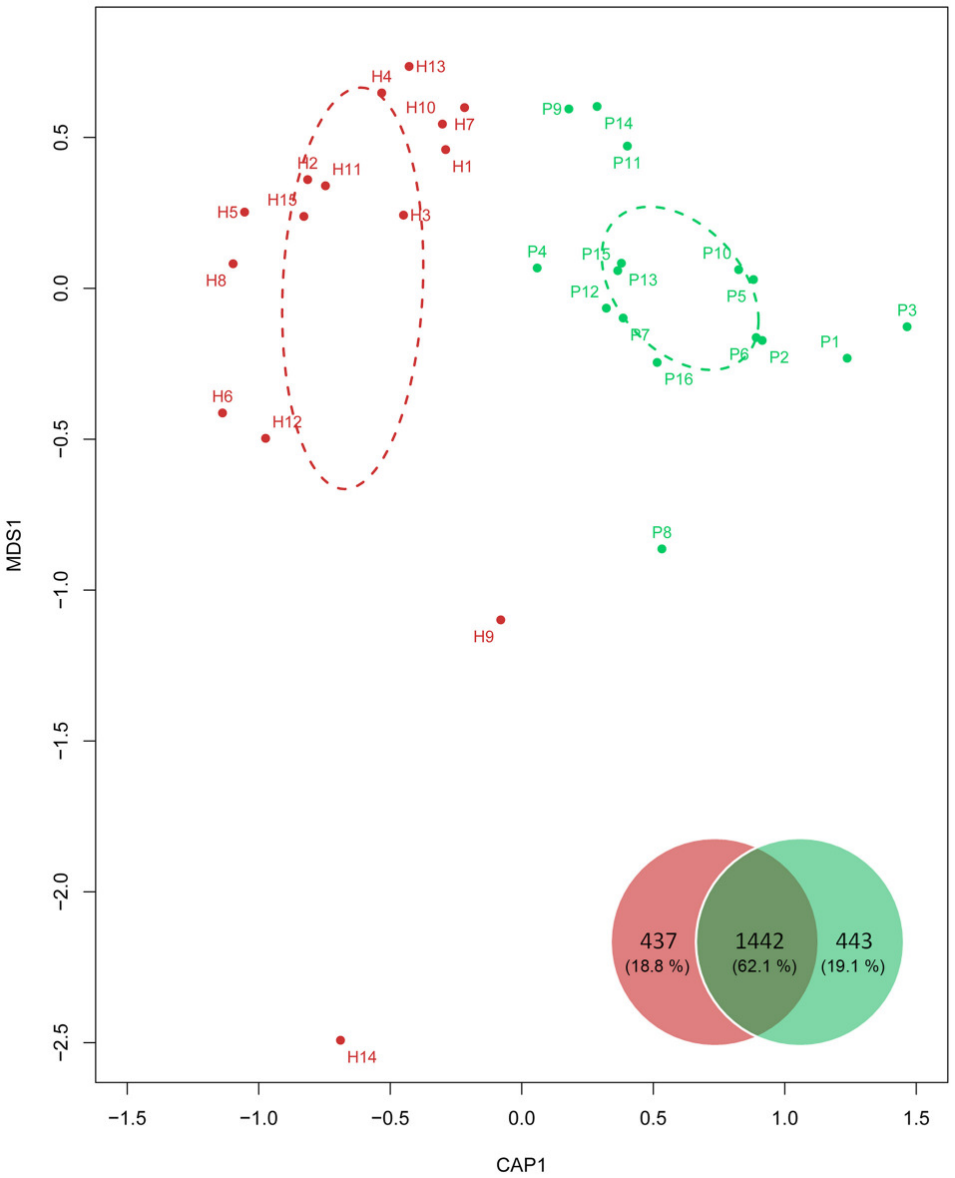
β-diversity comparison using constrained principal coordinate analysis (CAP) of unifrac distance across the 31 individuals. Dashed ellipses were drawn for the confidence areas (confidence limit = 0.95) of the healthy (red) and periodontitis (green) groups. The Venn diagram inset shows the numbers (percentages in all) of OTUs from the healthy (red) and periodontitis groups (green).

Following parallel sequencing of the 31 GCF samples, 150,990 high-quality reads were obtained. Clustering of all the qualified sequences at a 97% similarity level generated 2,322 OTUs, and more than 62% were shared by the healthy and periodontitis groups (Venn diagram inset in Figure 1). α*-*diversity was similar between individuals with and without periodontitis (*P* > 0.05). However, β-diversity comparisons displayed a significant difference between the healthy and periodontitis groups in CAP plots (Figure 1).

**Figure 2:**
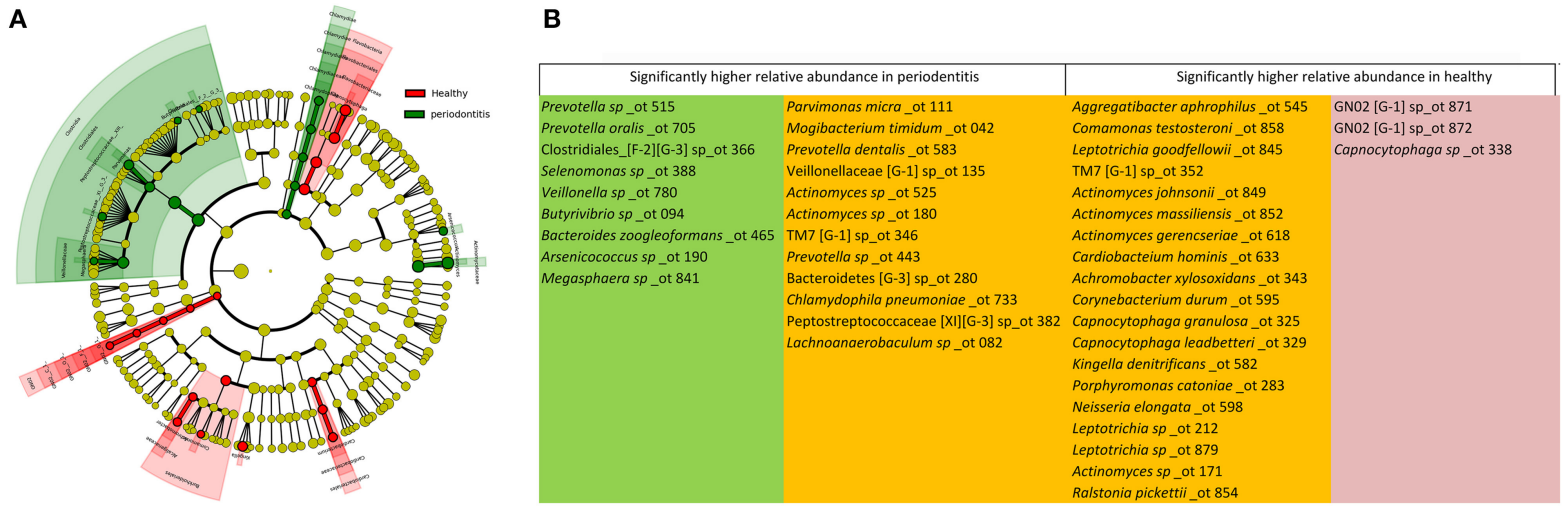
The dominant taxa in the healthy and periodontitis groups were analyzed using LEfSe (*P* < 0.05). **(A)** Visualization of differential taxa on a phylogenetic tree from phylum to genus level. **(B)** Significantly distinct species in the healthy and periodontitis groups. The green, red, and orange fields represent species found only in the periodontitis group, the healthy group, and in both, respectively.

The sequences were further divided into 13 phyla, 22 classes, 38 orders, 79 families, 154 genera, and 510 species. The majority of OTUs belonged to five major phyla (average ≥5%), including *Bacteroidetes, Firmicutes, Proteobacteria, Fusobacteria*, and *Actinobacteria* (Supplementary Table 2). The differences in the bacterial community composition were found at all taxonomic levels, including two minor phyla, four classes, six orders, eight families, 14 genera, and 43 species with significantly (*P* < 0.05) different relative abundance in the periodontitis group compared with the healthy group (Figure 2).

### Different bacterial distribution patterns in GCF of individuals with and without periodontitis

**Figure 3:**
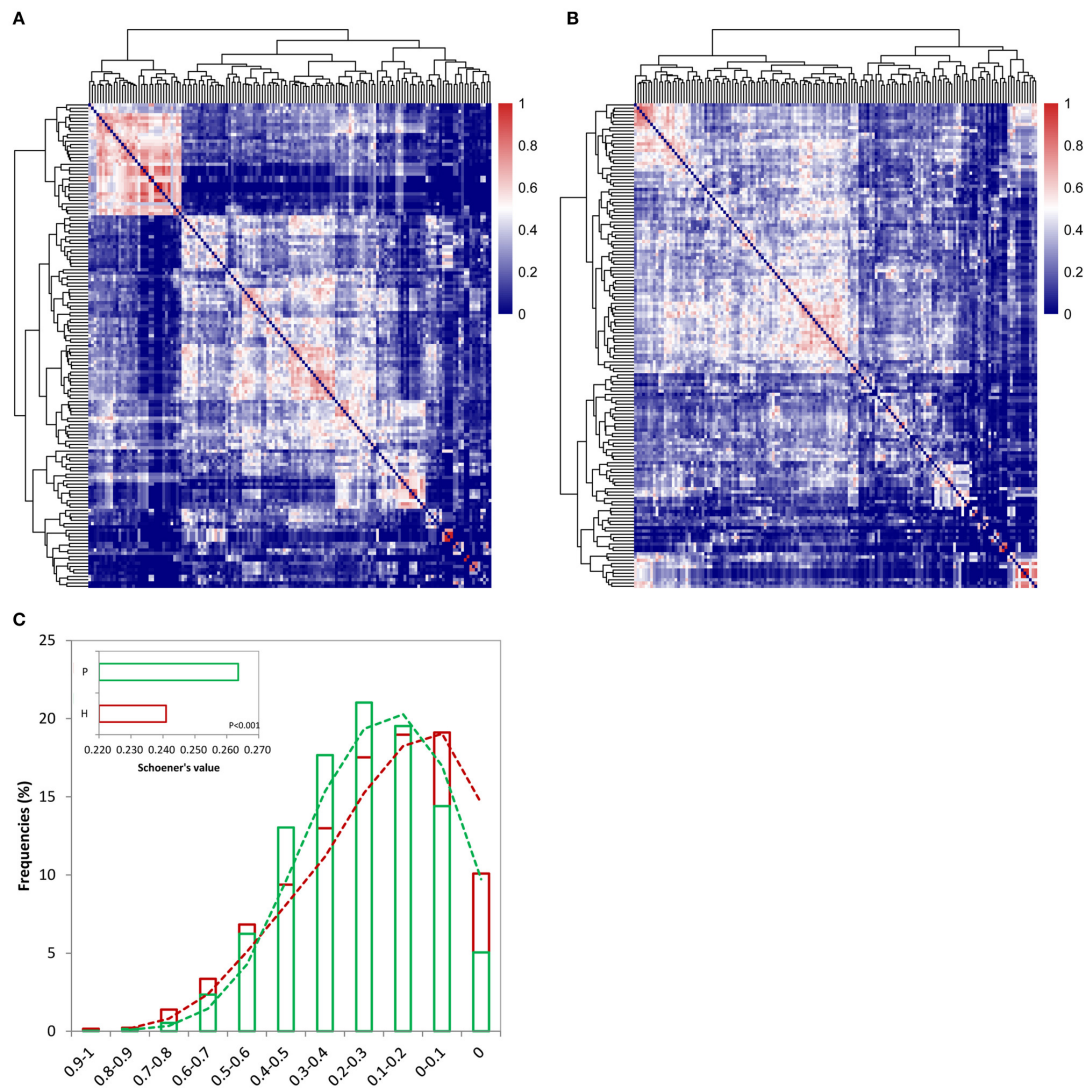
Heatmaps displaying genus distribution patterns for the healthy **(A)** and periodontitis **(B)** groups based on Schoener's index matrixes (Supplementary Tables 3, 4). Increasing values are translated into colors from blue to red. Trees were clustered based on the similarity of the Schoener's values. **(C)** The co-occurrence probabilities based on the Schoener's index were divided into 11 intervals, including 0, 0–0.1, 0.1–0.2, 0.2–0.3, 0.3–0.4, 0.4–0.5, 0.5–0.6, 0.6–0.7, 0.7–0.8, 0.8–0.9, and 0.9–1. The frequency of the Schoener's index falling into each interval was calculated in both the healthy (red) and periodontitis (green) groups. A global comparison of the Schoener's index between the two groups (using a *t*-test) is also shown within the plot.

To determine if there was a difference in the microbial communities between the GCF of individuals with and without periodontitis, Schoener's abundance-based co-occurrence probability analyses were performed for each group of genera. Two heatmaps (Figures 3A,B) from the resulting Schoener's index matrixes (Supplementary Tables 3, 4) were drawn to visually assess the bacterial structure within each group. All genera in each group were assembled into two clusters from the root of a dendrogram based on the similarity of their co-occurrence probabilities.

The analyses revealed a genera co-occurrence in the GCF of the periodontitis group compared to the GCF of the healthy group. For example, the genus *Peptostreptococcaceae*_XI_G_1 showed a significantly increased co-occurrence with other genera (*P* < 0.01). The Schoener's index values between *Peptostreptococcaceae*_XI_G_1 and *Bacillus* increased 3.7-fold in the GCF of individuals with periodontitis. However, the co-occurrence of the genus *Leptotrichiaceae*_G_1 with other genera significantly decreased (*P* < 0.01). The Schoener's index values between *Leptotrichiaceae*_G_1 and another two genera (*Leptotrichia* and *Aggregatibacter*) decreased 4.32- and 55-fold, respectively, in the GCF of individuals with periodontitis.

Furthermore, higher frequencies of extreme Schoener's value (i.e., ranging from 0.5 to 1 or ranging from 0 to 0.1) were detected from the healthy individuals compared with the periodontitis patients (Figure 3C), and a significantly higher value for Schoener's index was detected in the GCF from periodontitis patients (inset in Figure 3C, *P* < 0.001). These scenarios suggested that periodontitis was prone to high co-occurrence probabilities across more genera, and thereby probably generating different distribution patterns within the healthy and periodontitis groups.

The result demonstrates that polymicrobial community differed between the healthy and the periodontitis groups. The difference is attributed toward the co-occurrence of larger number of microbial communities at the genus level in the periodontitis group.

### Cytokines and their relations with the bacterial community structure in the GCF of samples with and without periodontitis

A difference is the cytokine composition was observed between GCF with and without periodontitis. Of the 20 cytokines examined (details in Supplementary Table 5), CRP was significantly (one-way ANOVA, *P* < 0.05) higher in the GCF of the periodontitis group while IL-8 was significantly (one-way ANOVA, *P* < 0.01) higher in the GCF of the healthy group.

**Figure 4:**
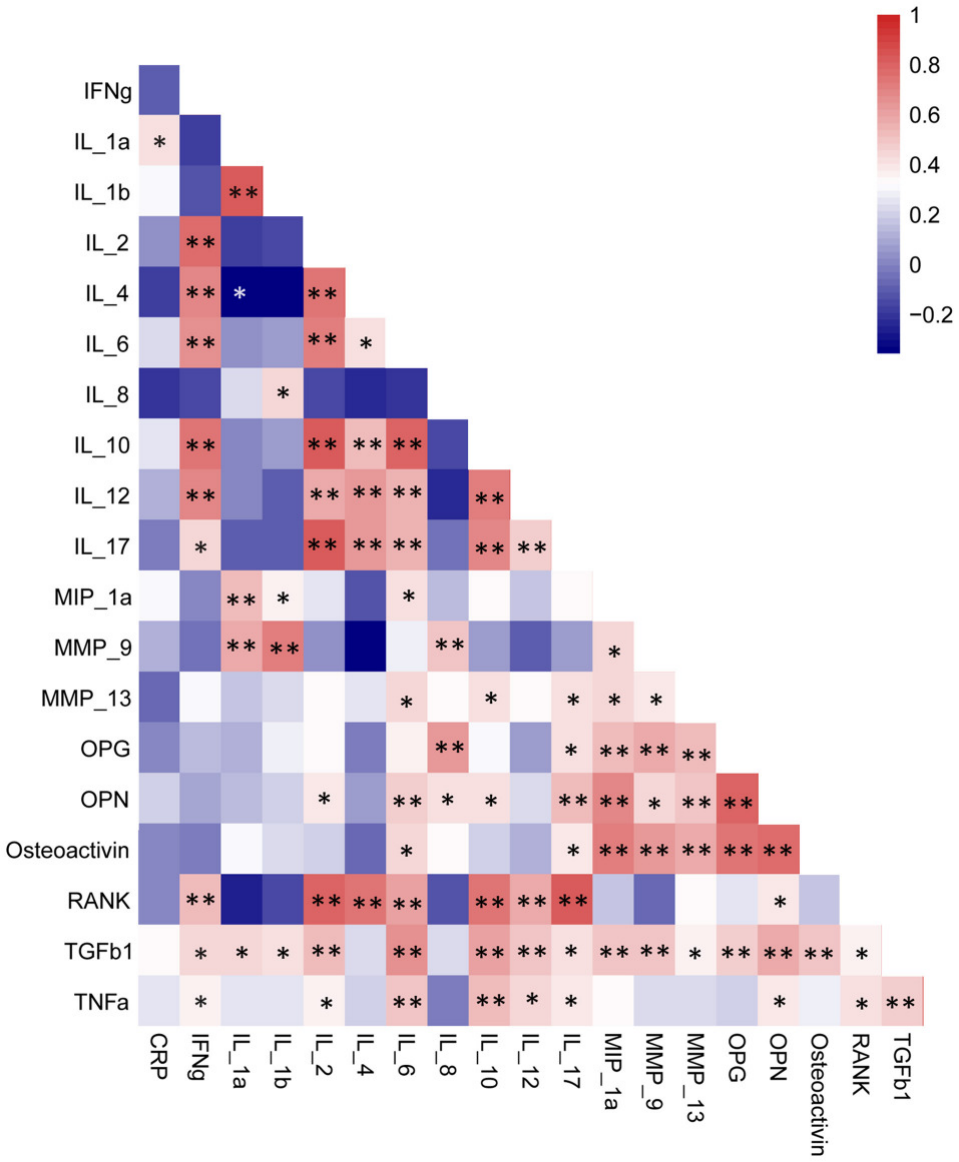
Correlations within the cytokines among all individuals. Increasing values are translated into colors from blue (negative correlation) to red (positive correlation). Significance: ^**^P<0.01; ^*^P<0.05. The data were detailed in Supplementary Table 6.

**Figure 5:**
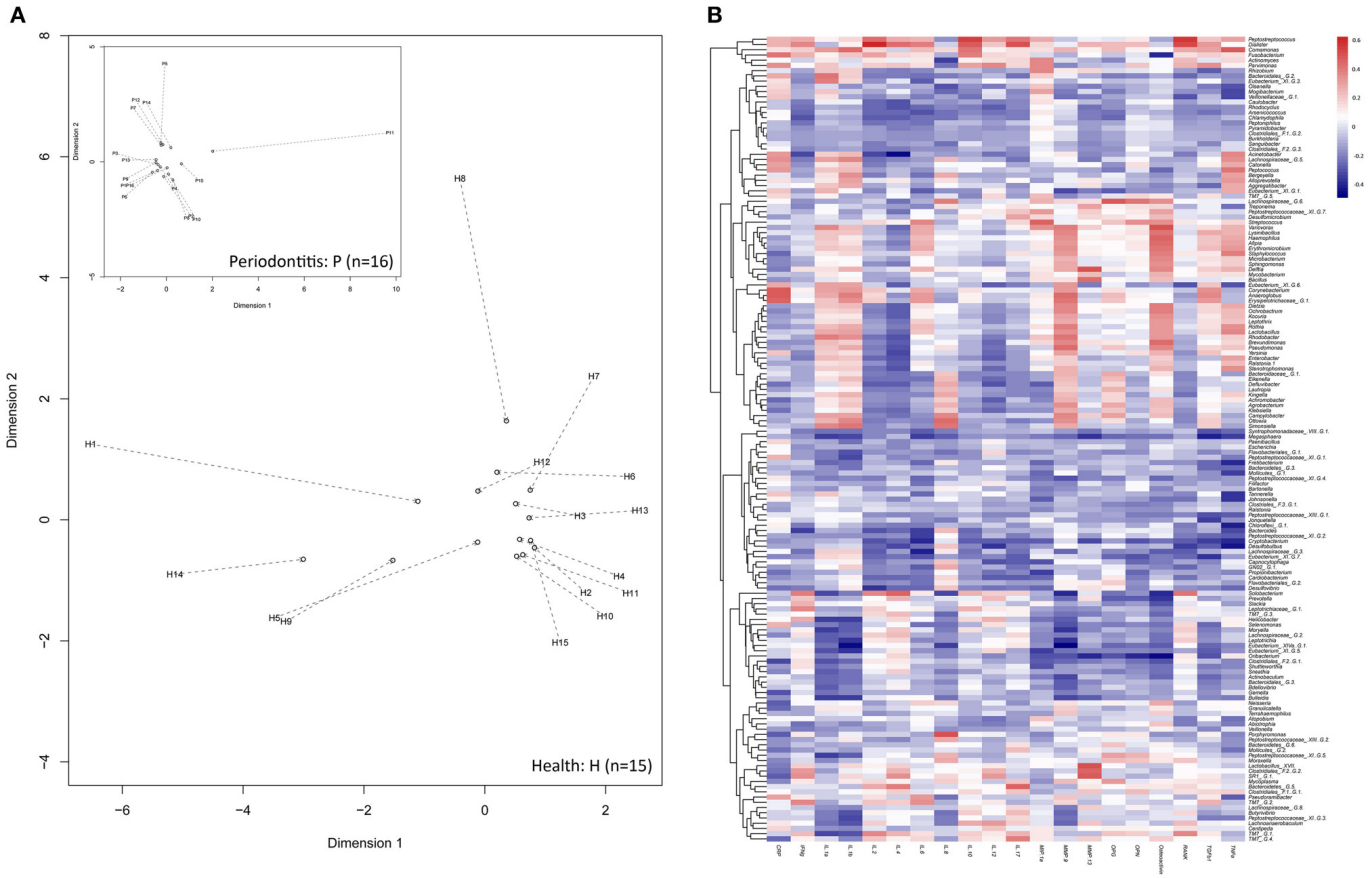
Associations between microbiomes and cytokines. **(A)** The Procrustean superimposition of individuals based on the UniFrac PCoAs of microbiomes (hollow circles) and cytokines (start point of the arrows) in the healthy group and the periodontitis group (inset plot). Dotted lines represent Procrustes residuals from both configurations. **(B)** Correlation matrices for cytokines and bacterial genera. Increasing values are translated into colors from blue (negative correlation) to red (positive correlation). Trees clustered based on the similarity of correlations between cytokines and genera.

Significant correlations were detected between pairs of cytokines (Figure 4, Supplementary Table 6), while variations in cytokines across the individuals could not be explained by the two groups, as we previously hypothesized (*P* > 0.1). Nevertheless, based on the distance matrix, the Mantel test revealed a significant concordance between the two data sets, i.e., between cytokines and microbiota, in the healthy group (Mantel statistic *r* = 0.36, *P* ≤ 0.05) rather than in the periodontitis group (Mantel statistic *r* = 0.013, *P* = 0.434). The results were further confirmed by the PROTEST test in the healthy (m_12_ = 0.61, *P* < 0.05) and periodontitis (m_12_ = 0.68, *P* = 0.933) groups, respectively. Configurations of cytokines could optimally fit to those of microbiomes in the healthy group but could not in the periodontitis group (Figure 5A).

To detect the specific effects, the correlations were also examined between cytokines and microbiomes at the genus level (Figure 5B, Supplementary Table 7). As shown in Table 1, MMP-9, osteoactivin, IL-8, and MIP-1a were significantly associated with bacterial composition at the phylum, class, order, family, and genus levels. CRP was also associated with bacterial composition at the species level, in addition to IL-8, MIP-1a, MMP-9, and osteoactivin. In turn, the bacterial taxa, from phylum to species levels, significantly influenced the cytokines, as listed in Table 2. The variation in cytokines communities could be significantly explained by 10 oral taxa belonging to five phyla.

**Table 1:** Effects of cytokines on the bacterial structure at different levels.

	**Phylum**		**Class**		**Order**		**Family**		**Genus**		**Species**	
	***r*^2^**	***P***	***r*^2^**	***P***	***r*^2^**	***P***	***r*^2^**	***P***	***r*^2^**	***P***	***r*^2^**	***P***
IL-8	0.2676	0.019^*^	0.3103	0.018^*^	0.2934	0.019^*^	0.2453	0.035^*^	0.1957	0.051.	0.2168	0.037^*^
MIP-1a	0.2104	0.043^*^	0.2231	0.027^*^	0.2622	0.013^*^	0.336	0.006^**^	0.3761	0.003^**^	0.5504	0.001^***^
MMP-9	0.2635	0.015^*^	0.2693	0.019^*^	0.2504	0.018^*^	0.2657	0.015^*^	0.2237	0.031^*^	0.4673	0.001^***^
Osteoactivin	0.2436	0.017^*^	0.3425	0.004^**^	0.3662	0.005^**^	0.3554	0.008^**^	0.362	0.007^**^	0.6423	0.001^***^
CRP	0.1453	0.091.	–	–	–	–	–	–	–	–	0.3337	0.005^**^

“^***^,” “^**^,” “^*^,” and “.” represent P < 0.001, 0.01, 0.05, and 0.1, respectively. –, represents that this variable was removed from the analysis.

**Table 2:** Effects of bacterial taxa on cytokines.

**Level**	**Taxa**	***r*^2^**	***P***
1	Firmicutes	0.1756	0.073.
1.1	Firmicutes|Clostridia	0.2914	0.004^**^
1.1.1	Firmicutes|Clostridia|Clostridiales	0.3307	0.011^*^
1.1.1.1	Firmicutes|Clostridia|Clostridiales|Peptostreptococcaceae_[XI]	0.2004	0.031^*^
1.1.1.2	Firmicutes|Clostridia|Clostridiales|Clostridiales_[F-2]	0.1616	0.067.
1.1.1.2.1	Firmicutes|Clostridia|Clostridiales|Clostridiales_[F-2]|Clostridiales_[F-2][G-1]	0.1573	0.088.
1.1.1.2.1.1	Firmicutes|Clostridia|Clostridiales|Clostridiales_[F-2]|Clostridiales_[F-2][G-1]|sp._oral_taxon_075	0.1573	0.085.
1.1.1.3	Firmicutes|Clostridia|Clostridiales|Veillonellaceae	0.0874	0.275
1.1.1.3.1	Firmicutes|Clostridia|Clostridiales|Veillonellaceae|Selenomonas	0.1622	0.08.
1.1.1.3.1.1	Firmicutes|Clostridia|Clostridiales|Veillonellaceae|Selenomonas|sputigena_oral_taxon_151	0.1762	0.068.
1.1.1.4	Firmicutes|Clostridia|Clostridiales|Peptostreptococcaceae_[XIII]	0.0333	0.65
1.1.1.4.1	Firmicutes|Clostridia|Clostridiales|Peptostreptococcaceae_[XIII]|Parvimonas	0.2144	0.04^*^
1.1.1.4.1.1	Firmicutes|Clostridia|Clostridiales|Peptostreptococcaceae_[XIII]|Parvimonas|micra_oral_taxon_111	0.2165	0.032^*^
2	Bacteroidetes	0.049	0.496
2.1	Bacteroidetes|Bacteroides	0.0249	0.71
2.1.1	Bacteroidetes|Bacteroides|Bacteroidales	0.0344	0.594
2.1.1.1	Bacteroidetes|Bacteroides|Bacteroidales|Porphyromonadaceae	0.0293	0.675
2.1.1.1.1	Bacteroidetes|Bacteroides|Bacteroidales|Porphyromonadaceae|Tannerella	0.0567	0.44
2.1.1.1.1.1	Bacteroidetes|Bacteroides|Bacteroidales|Porphyromonadaceae|Tannerella|sp._oral_taxon_286	0.1629	0.078.
3	Actinobacteria	0.0541	0.47
3.1	Actinobacteria|Actinobacteria	0.0523	0.489
3.1.1	Actinobacteria|Actinobacteria|Actinomycetales	0.0683	0.381
3.1.1.1	Actinobacteria|Actinobacteria|Actinomycetales|Corynebacteriaceae	0.2406	0.025^*^
3.1.1.1.1	Actinobacteria|Actinobacteria|Actinomycetales|Corynebacteriaceae|Corynebacterium	0.2406	0.02^*^
3.1.1.1.1.1	Actinobacteria|Actinobacteria|Actinomycetales|Corynebacteriaceae|Corynebacterium|matruchotii_oral_taxon_666	0.2377	0.023^*^
3.1.1.2	Actinobacteria|Actinobacteria|Actinomycetales|Micrococcaceae	0.2152	0.033^*^
3.1.1.2.1	Actinobacteria|Actinobacteria|Actinomycetales|Micrococcaceae|Rothia	0.2145	0.03^*^
3.1.1.3	Actinobacteria|Actinobacteria|Actinomycetales|Dietziaceae	0.1739	0.068.
3.1.1.3.1	Actinobacteria|Actinobacteria|Actinomycetales|Dietziaceae|Dietzia	0.1739	0.061.
3.1.1.3.1.1	Actinobacteria|Actinobacteria|Actinomycetales|Dietziaceae|Dietzia|sp._oral_taxon_368	0.1739	0.068.
3.1.1.3.1.2	Actinobacteria|Actinobacteria|Actinomycetales|Actinomycetaceae|Actinomyces|sp._oral_taxon_525	0.2061	0.042^*^
4	Fusobacteria	0.1929	0.05^*^
4.1	Fusobacteria|Fusobacteria	0.1931	0.055.
4.1.1	Fusobacteria|Fusobacteria|Fusobacteriales	0.2154	0.036^*^
4.1.1.1	Fusobacteria|Fusobacteria|Fusobacteriales|Fusobacteriaceae	0.1888	0.057.
4.1.1.1.1	Fusobacteria|Fusobacteria|Fusobacteriales|Fusobacteriaceae|Fusobacterium	0.1888	0.049^*^
4.1.1.1.1.1	Fusobacteria|Fusobacteria|Fusobacteriales|Fusobacteriaceae|Fusobacterium|nucleatum_ss_vincentii_oral_taxon_200	0.1481	0.085.
4.1.1.1.1.2	Fusobacteria|Fusobacteria|Fusobacteriales|Fusobacteriaceae|Fusobacterium|nucleatum_ss_animalis_oral_taxon_420	0.2007	0.045^*^
5	Proteobacteria	0.2595	0.02^*^
5.1	Proteobacteria|Gammaproteobacteria	0.2523	0.019^*^
5.1.1	Proteobacteria|Gammaproteobacteria|Pseudomonadales	0.2388	0.032^*^
5.1.1.1	Proteobacteria|Gammaproteobacteria|Pseudomonadales|Pseudomonadaceae	0.2322	0.027^*^
5.1.1.1.1	Proteobacteria|Gammaproteobacteria|Pseudomonadales|Pseudomonadaceae|Pseudomonas	0.2322	0.038^*^
5.1.1.1.1.1	Proteobacteria|Gammaproteobacteria|Pseudomonadales|Pseudomonadaceae|Pseudomonas|fluorescens_oral_taxon_612	0.2387	0.027^*^
5.2	Proteobacteria|Epsilonproteobacteria	0.1816	0.062.
5.2.1	Proteobacteria|Epsilonproteobacteria|Campylobacterales	0.1735	0.08.
5.2.1.1	Proteobacteria|Epsilonproteobacteria|Campylobacterales|Campylobacteraceae	0.164	0.089.
5.2.1.1.1	Proteobacteria|Epsilonproteobacteria|Campylobacterales|Campylobacteraceae|Campylobacter	0.164	0.094.

“^***^,” “^**^,” “^*^,” and “.” represent P < 0.001, 0.01, 0.05, and 0.1, respectively.

## Discussion

Understanding the interplay between the microbiome composition and its effects on the cytokines pattern could enable development of novel screening methods for periodontitis. Periodontal risk assessment is extremely important for clinical decision-making. The traditional periodontal diagnosis methods are based on clinical inspection and radiography, and they provide information only on the presence or absence of the disease (Taba et al., 2005). In order to develop a reliable diagnostic method for efficient clinical management, understanding the complex etiology of periodontal disease is imperative (Pihlstrom et al., 2005).

Therefore, the present study aimed to investigate whether there are differences in the combined profile of microbiome and cytokines in individuals with or without periodontitis. The results showed that a higher co-occurrence of genera in periodontitis group compared with the healthy group, as evaluated by Schoener's abundance-based co-occurrence index. The Mantel test revealed a significant concordance between cytokines and microbiota in the healthy group but not in the periodontitis group. Hence, alterations in the polymicrobial community structure leads to disruption in the healthy correlation between cytokines and microbiomes. This dysbiosis between the microbiota and the immune response could be one of the major etiological mechanisms underlying periodontitis. This study could allow the development of a scoring system that cold evaluate the risk of periodontitis in individuals.

The β-diversity comparisons revealed significant differences in the microbial community between the healthy and periodontitis groups. The relative abundance of 14 genera and 43 species was significantly different between the GCF of healthy individuals and individuals with periodontitis, but very few taxa concurred with those previously reported (Griffen et al., 2012; Abusleme et al., 2013; Kirst et al., 2015; Park et al., 2015). Abusleme et al. (2013) showed that microbiota from periodontitis had higher proportions of *Spirochetes, Synergistetes, Firmicutes*, and *Chloroflexi*, among other taxa, while the proportions of *Actinobacteria*, particularly *Actinomyces*, were higher in microbiota from healthy individuals; total *Actinomyces* load, however, remained constant between the two groups. Moreover, little overlap was detected with other study data. For example, *Porphyromonas endodontalis* and *P. gingivalis* are considered to be core species in periodontitis (Abusleme et al., 2013). However, *P. catoniae* was the only differentially enriched *Porphyromonas* species in the present study, with abundance being higher in healthy individuals. Consistent with our finding, previous studies have shown that *P. catoniae* was specifically associated with healthy sites rather than diseased deep pockets (de Lillo et al., 2004; Crielaard et al., 2011). Therefore, the species causing periodontitis are various and depend on various factors such as genetics (both bacterial and host), antibiotics, diet, and carbohydrate types (Khan et al., 2015). Discrepancies among studies could be due to these factors, as well as to the methods used to evaluate the microbiota. To overcome the biases that could be introduced by different habitats, only individuals having very similar living habits were included. In addition, recent antibiotic treatment led to exclusion. The present study used a combination of two high-throughput methods (454 pyrosequencing and RayBio Quantibody Arrays) for identifying the bacteria and cytokines, which is an innovation compared to previous studies (Griffen et al., 2012; Abusleme et al., 2013; Kirst et al., 2015; Park et al., 2015).

Obviously, some microorganisms detected in the present study are pathogens while some other are commensal, are their roles could be different in different environments. The observations made in this study from the Schoener's abundance-based co-occurrence probability analysis revealed different distribution patterns in subjects with periodontitis and healthy subjects, similar to what is observed in oral caries (Zhou et al., 2016). When the microbial community structure of the GCF is disrupted (Lamont and Hajishengallis, 2015), there is a transition toward a competitive pattern that may lead to periodontitis. In addition, each component of a biological system affects the other components. Additional studies are necessary to determine the exact inter-relationships among the microorganisms detected here.

The exact dynamic evolution of the bacterial communities during the development of periodontitis is unknown. As stated above, the microbial communities are different among healthy sites and shallow and deep pockets of periodontitis (Abusleme et al., 2013), but how these communities evolve in time is currently unknown. Studies should be designed to determine whether pathogenic bacteria invade susceptibility sites and repel commensal bacteria, or if these pathogenic bacteria are already present and cause the disease following a decrease in host defenses (Darveau, 2010). Nevertheless, the disruption of the host response to the microbial communities is involved in periodontitis and it may be responsible, at least in part, for the progression of healthy gingival sites to gingivitis and periodontitis (Darveau, 2010). Furthermore, in addition to disrupted host response, keystone pathogens that increase community virulence will promote the progression of periodontitis (Lamont and Hajishengallis, 2015).

Microbial dysbiosis is involved in altered host-microbial interaction (Bartold and Van Dyke, 2013). In the present study, significantly higher levels of CRP were observed in the periodontitis group while IL-8 was higher in the healthy group, but these results are different from those of previous studies (Cheng et al., 2014). Lagdive et al. (2013) showed that IL-8 levels were higher in the GCF of patients with periodontitis. Shaddox et al. (2011) studies diseased and healthy sites within the same individuals and showed that healthy sites had higher IL-8 levels than diseased ones, supported by Teles et al. (2010). Shojaee et al. (2013) showed that the saliva of patients with periodontitis had higher levels of CRP compared to controls. Wohlfeil et al. (2012) showed that CRP was elevated in the saliva of patients with aggressive periodontitis, while IL-8 was similar compared with controls. These inconsistencies among different studies may be attributed to different study methods and the inherent biological diversity between individuals living in different environments, i.e., with different diets, carbohydrate intake, and stress (Seow, 2012; Cheng et al., 2014). Thus, whether these biomarkers are clinically significant is debatable, and they require further validation in different populations (Cheng et al., 2014). Therefore, once again, considering only the GCF cytokines is not better than considering only the microbiome, and an assessment of the two should provide a better indicator for the development of periodontitis. Nevertheless, comprehensive profiling is currently expensive and can only be done in a research context.

In this study, a significant association was detected between the microbiome and the GCF cytokines. There is a reciprocal genetic and epigenetic crosstalk between the microbiota and the host immune response (Levy et al., 2015). Therefore, during the observed microbial shifts, cytokines, particularly IL-8 and CRP, might have a significant influence on the overall microbial community. As observed in this study and by Shojaee et al. (2013), the CRP levels are significantly higher in patients with periodontal disease compared to healthy subjects. The shift in CRP and other cytokines can be attributed to the shift in the composition of the microbial community affecting the inflammatory reaction in the host. Given the assembly of a heterotypic community by compatible organisms in a gingival crevice (Hajishengallis and Lamont, 2012; Hajishengallis, 2014), the microbiome may also significantly affect the cytokine levels, particularly for *Parvimonas micra* oral taxon 111, *Corynebacterium matruchotii* oral taxon 666, *Actinomyces* sp. oral taxon 525, *Fusobacterium nucleatum* oral taxon 420, and *Pseudomonas fluorescens* oral taxon 621. Therefore, rather than individual healthy- and/or pathogen-related microbes, whole ecological shifts (Abusleme et al., 2013; Schwarzberg et al., 2014), particularly the conversion of the community structure pattern and the correlation between cytokines and microbiomes, may be the major etiological mechanism underlying periodontitis, as suggested by the present study.

In the present study, α-diversity and community-level analyses (UniFrac-based PCoA) did not identify clear differences between the GCF from healthy individuals and that from individuals with periodontitis. One possible explanation is that all individuals in this study were from the same living area, which could result in non-significant differences of bacterial α-diversity between the healthy and the periodontitis groups due to the highly similar diet and life habits. Additionally, the major phyla were similar to those identified in previous studies (Griffen et al., 2012; Abusleme et al., 2013; Kirst et al., 2015; Park et al., 2015), which suggests that the common phyla in the GCF are relatively constant.

Nevertheless, this study is not without limitation. Indeed, the sample size was small and from a single geographical region. The samples were pooled. The cytokine analysis focused on 20 selected cytokines. Further investigation with a larger sample size distributed across a wide geographical area may be required for better understanding of the microbiome composition and its influence on cytokine composition in periodontitis patients. In addition, improvements in the high-flux quantitative detection of cytokines should enable expansive cytokinomics in the future to uncover additional underlying issues.

In conclusion, alterations in the polymicrobial community structure leads to disruption in the healthy correlation between cytokines and microbiomes. This dysbiosis between the microbiota and the immune response could be one of the major etiological mechanisms underlying periodontitis. These results provide a new link between microbiota and cytokines that will help developing strategies for the diagnosis, prognosis, and efficient monitoring of periodontal therapy.

## Author contributions

JZ, NJ, and ZL provided the funding. JZ, YY, KJ, NJ, ZY, and ZL conceived and designed the study. JZ, XH, and XZ contributed to the acquisition of GCF samples. FW and JL participated in the experiments. NJ and GZ analyzed the sequencing data. JZ and YY wrote the draft of the manuscript, NJ and ZL helped to revise it. All authors reviewed the manuscript.

### Conflict of interest statement

The authors declare that the research was conducted in the absence of any commercial or financial relationships that could be construed as a potential conflict of interest.
