# Supplementary material for: Relationship between Gingival Crevicular Fluid Microbiota and Cytokine Profile in Periodontal Host Homeostasis
Source: Front Microbiol. 2017 Nov 1;8:2144. doi: 10.3389/fmicb.2017.02144 (PMC5672786; doi:10.3389/fmicb.2017.02144)
Supplement: Supplementary file 1 [file Table1.DOCX]

**Supplementary Table S1.** Characteristics of the 31 individuals in this study.

| Group | Age (mean ± SD) | Gender female, n (%) | BOP (%) | PD (mean ± SD) | CAL (mean ± SD) |
| --- | --- | --- | --- | --- | --- |
| Periodontitis (N=16) | 49.1 ± 8.6 | 7 (44%) | ≥30 * | 6.4 ± 1.1* | 3.4 ± 1.3* |
| Healthy subjects (N=15) | 36.7 ± 9.1 | 7 (47%) | <30 | 3.0 ± 0.1 | 0 |

Abbreviations: SD, standard deviation of the mean; BOP, bleeding on probing; PD, pocket probing depths; CAL, clinical attachment loss. PD and CAL were measured in mm. Asterisks indicate significant differences between periodontitis and healthy groups (P < 0.0001).
