# Supplementary material for: Relationship between Gingival Crevicular Fluid Microbiota and Cytokine Profile in Periodontal Host Homeostasis
Source: Front Microbiol. 2017 Nov 1;8:2144. doi: 10.3389/fmicb.2017.02144 (PMC5672786; doi:10.3389/fmicb.2017.02144)
Supplement: Supplementary file 2 [file Table2.DOCX]

**Supplementary Table S2.** Taxonomic resolutions (relative abundance %) for the bacterial sequences in the groups with and without periodontitis at the phylum, class, order, and family levels

|  |  | Health | Periodontitis |
| --- | --- | --- | --- |
| 1 | Bacteroidetes | 28.48±10.84 | 24.03±9.67 |
| 1.1 | Bacteroides | 22.78±10.44 | 19.00±6.29 |
| 1.1.1 | Bacteroidales | 22.78±10.44 | 19.00±6.29 |
| 1.1.1.1 | Bacteroidaceae | 0.54±1.37 | 0.47±0.41 |
| 1.1.1.2 | Bacteroidales_[F-2] | 0.26±0.27 | 0.50±0.8 |
| 1.1.1.3 | Bacteroidales_[F-3] | 0.00±0.01 | 0.02±0.05 |
| 1.1.1.4 | Porphyromonadaceae | 11.04±7.62 | 7.68±4.75 |
| 1.1.1.5 | Prevotellaceae | 10.95±5.47 | 10.33±3.82 |
| 1.2 | Bacteroidetes_[C-1] | 1.81±3.11 | 3.44±6.25 |
| 1.2.1 | Bacteroidetes_[O-1] | 1.81±3.11 | 3.44±6.25 |
| 1.2.1.1 | Bacteroidetes_[F-1] | 1.81±3.11 | 3.44±6.25 |
| 1.3 | Bacteroidetes_[C-2] | 0.14±0.32 | 0.24±0.5 |
| 1.3.1 | Bacteroidetes_[O-2] | 0.14±0.32 | 0.24±0.5 |
| 1.3.1.1 | Bacteroidetes_[F-2] | 0.14±0.32 | 0.24±0.5 |
| 1.4 | Flavobacteria | 3.75±3.85 | 1.36±1.59 |
| 1.4.1 | Flavobacteriales | 3.75±3.85 | 1.36±1.59 |
| 1.4.1.1 | Flavobacteriaceae | 3.73±3.85 | 1.30±1.6 |
| 1.4.1.2 | Flavobacteriales_[F-1] | 0.01±0.02 | 0.06±0.11 |
| 1.4.1.3 | Flavobacteriales_[F-2] | 0.00±0.01 | 0.00±0.01 |
| 2 | Firmicutes | 23.48±5.49 | 29.16±9.05 |
| 2.1 | Bacilli | 12±5.65 | 13.87±9.84 |
| 2.1.1 | Bacillales | 0.81±0.68 | 0.74±0.78 |
| 2.1.1.1 | Bacillaceae | 0.05±0.1 | 0.03±0.06 |
| 2.1.1.2 | Paenibacillaceae | 0.00±0.01 | 0.01±0.02 |
| 2.1.1.3 | Staphylococcaceae | 0.76±0.64 | 0.7±0.75 |
| 2.1.2 | Lactobacillales | 11.19±5.15 | 13.13±9.95 |
| 2.1.2.1 | Aerococcaceae | 0.05±0.08 | 0.11±0.26 |
| 2.1.2.2 | Carnobacteriaceae | 0.85±0.86 | 0.68±0.83 |
| 2.1.2.3 | Lactobacillaceae | 0.11±0.21 | 0.04±0.04 |
| 2.1.2.4 | Streptococcaceae | 10.17±4.77 | 12.29±9.3 |
| 2.2 | Clostridia | 11.02±4.55 | 14.84±3.34 |
| 2.2.1 | Clostridiales | 11.02±4.55 | 14.84±3.34 |
| 2.2.1.1 | Clostridiales_[F-1] | 0.05±0.09 | 0.06±0.12 |
| 2.2.1.2 | Clostridiales_[F-2] | 0.86±1.14 | 0.53±1.08 |
| 2.2.1.3 | Clostridiales_[F-3] | 0.01±0.02 | 0 |
| 2.2.1.4 | Eubacteriaceae_[XV] | 0.03±0.05 | 0.09±0.12 |
| 2.2.1.5 | Lachnospiraceae_[XIVa] | 2.37±1.42 | 2.07±1.42 |
| 2.2.1.6 | Peptococcaceae | 0.48±0.53 | 0.38±0.55 |
| 2.2.1.7 | Peptostreptococcaceae_[XIII] | 0.5±0.87 | 0.92±0.7 |
| 2.2.1.8 | Peptostreptococcaceae_[XI] | 2.66±1.43 | 4.24±2.2 |
| 2.2.1.9 | Syntrophomonadaceae_[VIII] | 0.01±0.03 | 0.09±0.14 |
| 2.2.1.10 | Veillonellaceae | 4.04±2.46 | 6.46±2.92 |
| 2.3 | Erysipelotrichi | 0.45±0.47 | 0.46±0.43 |
| 2.3.1 | Erysipelotrichales | 0.35±0.34 | 0.42±0.4 |
| 2.3.1.1 | Erysipelotrichaceae | 0.35±0.34 | 0.42±0.4 |
| 2.3.2 | Erysipelotrichi_[O-1] | 0.11±0.2 | 0.04±0.07 |
| 2.3.2.1 | Erysipelotrichi_[F-1] | 0.11±0.2 | 0.04±0.07 |
| 3 | Proteobacteria | 15.78±13.43 | 9.25±5.77 |
| 3.1 | Alphaproteobacteria | 0.56±1.14 | 0.35±0.41 |
| 3.1.1 | Caulobacterales | 0.08±0.15 | 0.08±0.11 |
| 3.1.1.1 | Proteobacteria\|Alphaproteobacteria\|Caulobacterales\|Caulobacteraceae | 0.08±0.15 | 0.08±0.11 |
| 3.1.2 | Rhizobiales | 0.36±0.73 | 0.19±0.23 |
| 3.1.2.1 | Bartonellaceae | 0.03±0.1 | 0.04±0.1 |
| 3.1.2.2 | Bradyrhizobiaceae | 0.1±0.33 | 0.04±0.05 |
| 3.1.2.3 | Brucellaceae | 0.18±0.38 | 0.06±0.09 |
| 3.1.2.4 | Phyllobacteriaceae | 0.03±0.05 | 0.01±0.02 |
| 3.1.2.5 | Rhizobiaceae | 0.02±0.04 | 0.03±0.06 |
| 3.1.3 | Rhodobacterales | 0.01±0.02 | 0.01±0.02 |
| 3.1.3.1 | Rhodobacteraceae | 0.01±0.02 | 0.01±0.02 |
| 3.1.4 | Sphingomonadales | 0.1±0.26 | 0.07±0.16 |
| 3.1.4.1 | Sphingomonadaceae | 0.1±0.26 | 0.07±0.16 |
| 3.2 | Betaproteobacteria | 8.05±7.06 | 4.41±5.79 |
| 3.2.1 | Burkholderiales | 1.26±1.56 | 0.34±0.33 |
| 3.2.1.1 | Alcaligenaceae | 0.12±0.15 | 0.01±0.01 |
| 3.2.1.2 | Burkholderiaceae | 0.79±1.21 | 0.24±0.34 |
| 3.2.1.3 | Comamonadaceae | 0.11±0.17 | 0.03±0.05 |
| 3.2.1.4 | Comomonadaceae | 0.05±0.12 | 0.02±0.03 |
| 3.2.1.5 | Ralstoniaceae | 0.19±0.32 | 0.04±0.07 |
| 3.2.2 | Neisseriales | 6.79±6.03 | 4.03±5.64 |
| 3.2.2.1 | Neisseriaceae | 6.79±6.03 | 4.03±5.64 |
| 3.2.3 | Rhodocyclales | 0.01±0.02 | 0.04±0.07 |
| 3.2.3.1 | Rhodocyclaceae | 0.01±0.02 | 0.04±0.07 |
| 3.3 | Deltaproteobacteria | 0.47±0.73 | 0.87±1.46 |
| 3.3.1 | Bdellovibrionales | 0.01±0.02 | 0.03±0.1 |
| 3.3.1.1 | Bdellovibrionacae | 0.01±0.02 | 0.03±0.1 |
| 3.3.2 | Desulfobacterales | 0.24±0.32 | 0.17±0.19 |
| 3.3.2.1 | Desulfobulbaceae | 0.24±0.32 | 0.17±0.19 |
| 3.3.3 | Desulfovibrionales | 0.23±0.48 | 0.67±1.35 |
| 3.3.3.1 | Desulfomicrobiaceae | 0.15±0.36 | 0.62±1.34 |
| 3.3.3.2 | Desulfovibrionaceae | 0.08±0.13 | 0.05±0.08 |
| 3.4 | Epsilonproteobacteria | 1.24±1.43 | 0.90±0.76 |
| 3.4.1 | Campylobacterales | 1.24±1.43 | 0.90±0.76 |
| 3.4.1.1 | Campylobacteraceae | 1.23±1.43 | 0.87±0.76 |
| 3.4.1.2 | Helicobacteraceae | 0.01±0.01 | 0.02±0.03 |
| 3.5 | Gammaproteobacteria | 5.46±5.57 | 2.72±2.09 |
| 3.5.1 | Cardiobacteriales | 0.26±0.4 | 0.07±0.11 |
| 3.5.1.1 | Cardiobacteriaceae | 0.26±0.4 | 0.07±0.11 |
| 3.5.2 | Enterobacteriales | 0.07±0.11 | 0.04±0.05 |
| 3.5.2.1 | Enterobacteriaceae | 0.07±0.11 | 0.04±0.05 |
| 3.5.3 | Pasteurellales | 2.23±1.97 | 1.55±1.85 |
| 3.5.3.1 | Pasteurellaceae | 2.23±1.97 | 1.55±1.85 |
| 3.5.4 | Pseudomonadales | 2.86±4.84 | 1.05±0.9 |
| 3.5.4.1 | Moraxellaceae | 0.28±0.39 | 0.37±0.38 |
| 3.5.4.2 | Pseudomonadaceae | 2.58±4.67 | 0.68±0.65 |
| 3.5.5 | Xanthomonadales | 0.03±0.08 | 0.02±0.04 |
| 3.5.5.1 | Xanthomonadaceae | 0.03±0.08 | 0.02±0.04 |
| 4 | Fusobacteria | 13.45±7.62 | 10.94±5.21 |
| 4.1 | Fusobacteria | 13.45±7.62 | 10.94±5.21 |
| 4.1.1 | Fusobacteriales | 13.45±7.62 | 10.94±5.21 |
| 4.1.1.1 | Fusobacteriaceae | 6.34±3.11 | 5.93±4.06 |
| 4.1.1.2 | Leptotrichiaceae | 7.10±6.66 | 5.01±4.38 |
| 5 | Actinobacteria | 12.54±5.2 | 17.33±10.09 |
| 5.1 | Actinobacteria | 12.54±5.2 | 17.33±10.09 |
| 5.1.1 | Actinomycetales | 11.87±5.14 | 16.47±10.13 |
| 5.1.1.1 | Actinomycetaceae | 7.13±2.69 | 12.31±8.02 |
| 5.1.1.2 | Corynebacteriaceae | 2.45±2.74 | 2.92±5.1 |
| 5.1.1.3 | Dietziaceae | 0.73±1.59 | 0.32±0.38 |
| 5.1.1.4 | Microbacteriaceae | 0.02±0.04 | 0.01±0.03 |
| 5.1.1.5 | Micrococcaceae | 0.62±1.62 | 0.61±1.2 |
| 5.1.1.6 | Mycobacteriaceae | 0.01±0.01 | 0.01±0.01 |
| 5.1.1.7 | Propionibacteriaceae | 0.93±1.43 | 0.27±0.35 |
| 5.1.1.8 | Sanguibacteraceae | 0.00±0.02 | 0.03±0.05 |
| 5.1.2 | Coriobacteriales | 0.67±1.11 | 0.86±1.02 |
| 5.1.2.1 | Coriobacteriaceae | 0.67±1.11 | 0.86±1.02 |
| 6 | TM7 | 3.39±2.44 | 5.64±3.96 |
| 6.1 | TM7_[C-1] | 3.39±2.44 | 5.64±3.96 |
| 6.1.1 | TM7_[O-1] | 3.39±2.44 | 5.64±3.96 |
| 6.1.1.1 | TM7_[F-1] | 2.61±1.52 | 3.65±2.19 |
| 6.1.1.2 | TM7_[F-2] | 0.78±1.17 | 1.98±2.95 |
| 7 | Synergistetes | 1.21±1.59 | 1.31±1.47 |
| 7.1 | Synergistetes_[C-1] | 1.21±1.59 | 1.31±1.47 |
| 7.1.1 | Synergistetes_[O-1] | 1.21±1.59 | 1.31±1.47 |
| 7.1.1.1 | Synergistetes_[F-1] | 0.00±0.01 | 0.05±0.14 |
| 7.1.1.2 | Synergistetes_[F-2] | 1.21±1.59 | 1.26±1.49 |
| 8 | Spirochaetes | 1.14±1.12 | 1.62±1.18 |
| 8.1 | Spirochaetes | 1.14±1.12 | 1.62±1.18 |
| 8.1.1 | Spirochaetales | 1.14±1.12 | 1.62±1.18 |
| 8.1.1.1 | Spirochaetaceae | 1.14±1.12 | 1.62±1.18 |
| 9 | Tenericutes | 0.20±0.31 | 0.14±0.14 |
| 9.1 | Mollicutes | 0.20±0.31 | 0.14±0.14 |
| 9.1.1 | Mollicutes_[O-1] | 0.01±0.03 | 0.03±0.06 |
| 9.1.1.1 | Mollicutes_[F-1] | 0.01±0.03 | 0.03±0.06 |
| 9.1.2 | Mollicutes_[O-2] | 0.02±0.03 | 0.03±0.06 |
| 9.1.2.1 | Mollicutes_[F-2] | 0.02±0.03 | 0.03±0.06 |
| 9.1.3 | Mycoplasmatales | 0.17±0.29 | 0.07±0.08 |
| 9.1.3.1 | Mycoplasmataceae | 0.17±0.29 | 0.07±0.08 |
| 10 | SR1 | 0.19±0.31 | 0.11±0.27 |
| 10.1 | SR1_[C-1] | 0.19±0.31 | 0.11±0.27 |
| 10.1.1 | SR1_[O-1] | 0.19±0.31 | 0.11±0.27 |
| 10.1.1.1 | SR1_[F-1] | 0.19±0.31 | 0.11±0.27 |
| 11 | Chloroflexi | 0.13±0.12 | 0.29±0.25 |
| 11.1 | Chloroflexi_[C-1] | 0.13±0.12 | 0.29±0.25 |
| 11.1.1 | Chloroflexi_[O-1] | 0.13±0.12 | 0.29±0.25 |
| 11.1.1.1 | Chloroflexi_[F-1] | 0.13±0.12 | 0.29±0.25 |
| 12 | GN02 | 0.02±0.03 | 0 |
| 12.1 | GN02_[C-1] | 0.02±0.03 | 0 |
| 12.1.1 | GN02_[O-1] | 0.02±0.03 | 0 |
| 12.1.1.1 | GN02_[F-1] | 0.02±0.03 | 0 |
| 13 | Chlamydiae | 0.01±0.02 | 0.18±0.26 |
| 13.1 | Chlamydiae | 0.01±0.02 | 0.18±0.26 |
| 13.1.1 | Chlamydiales | 0.01±0.02 | 0.18±0.26 |
| 13.1.1.1 | Chlamydiaceae | 0.01±0.02 | 0.18±0.26 |
